# Giant Cell Tumor of Tendon Sheath in Knee Capsule

**DOI:** 10.7759/cureus.16632

**Published:** 2021-07-26

**Authors:** Bader Alghamdi, Saeed Koaban, Hossam Alnaqa

**Affiliations:** 1 Orthopaedics, Prince Mohammad Bin Abdulaziz Hospital, Riyadh, SAU; 2 Orthopaedic Surgery, Security Forces Hospital, Riyadh, SAU

**Keywords:** giant cell tumor, tendon sheath

## Abstract

A giant cell tumor of the tendon sheath (GCTTS) is a benign, soft tissue (synovial membrane) tumor that frequently involves the fingers. However, the localized form of the GCTTS can be rarely seen in large joints such as knees, ankles, and shoulders. GCTTS may occur due to questionable etiology that includes inflammation, trauma, or chromosomal abnormalities. This is a case of a 30-year-old female, who presented with a painful mass in her right knee. Examination revealed a palpable, tender, slightly mobile mass lateral to the patella. MRI demonstrated a cystic lesion that underwent excision. Data from the reported cases imply that local recurrence was observed in 10%-20% of patients due to incomplete primary resection. Physicians should consider the likelihood of a GCTTS when an intra-articular knee prominence is found. Early diagnosis and appropriate treatment can help with rapid clinical improvement.

## Introduction

A giant cell tumor of the tendon sheath (GCTTS) is a benign proliferation of synovial lining tissues in joints, bursa, and tendon sheath. Previous literature has described it using many names, such as a tenosynovial giant cell tumor or pigmented villonodular synovitis (PVNS) [1-2]. These variations are identical in histology but differ in how the disease presents itself [3-4]. For instance, a GCTTS is found mainly in extra-articular regions within flexor tendons of the hands and feet and is considered to be a localized form. However, PVNS is a more rare and diffuse form, in intra-articular regions and involves large joints like knees, ankles, and shoulders [3]. A retrospective study of 207 GCTTS patients revealed that only 4% of cases involved the knee [5]. We present a rare case of local GCTTS affecting the capsule of the knee, and discuss its clinical and radiographic characteristics, diagnosis, and management.

## Case presentation

**Figure 1:**
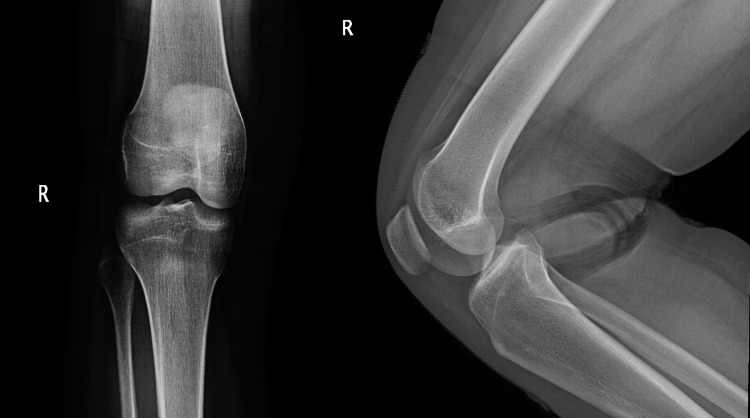
Anteroposterior and lateral right knee radiographs showed no bony abnormality.

**Figure 2:**
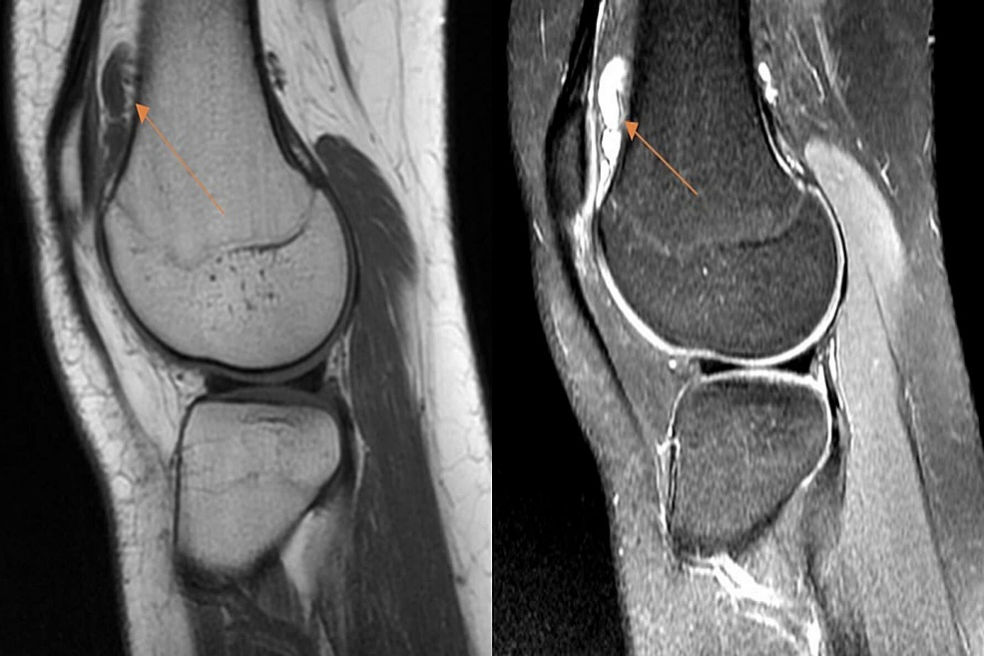
Hypointense lesion on T1-weighted MRI sequences and hyperintense lesion on T2-weighted MRI sequence show a cystic lesion within the pre-femoral fat.

A 30-year-old woman presented to our clinic with the complaint of non-specific right knee pain, which had been gradually increasing in severity over the past three years. The pain had been associated with the feeling of a mass in her lateral joint line, progressing and enlarging in size. The mass was restricting her knee movement. She also reported occasional clicking and locking of her knee during the past year, without her knee giving way. There was no history of trauma to the knee, with no relevant medical or surgical history. A physical examination showed a palpable, slightly mobile lobulated 2.0 cm × 1.0 cm mass lateral to the patella. The knee had a full range of motion and mild tenderness. No signs of meniscal or ligamentous injury were found. The neurovascular examination was unremarkable. Plain radiographs showed no abnormality as shown in Figure 1. MRI showed a cystic lesion within the pre-femoral fat on the lateral side, measuring 2 cm x 1.6 cm x 0.7 cm. The lesion’s characteristics suggested a ganglion cyst. The lesion was hypointense in T1 images and hyperintense in T2 images shown in Figure 2.

**Figure 3:**
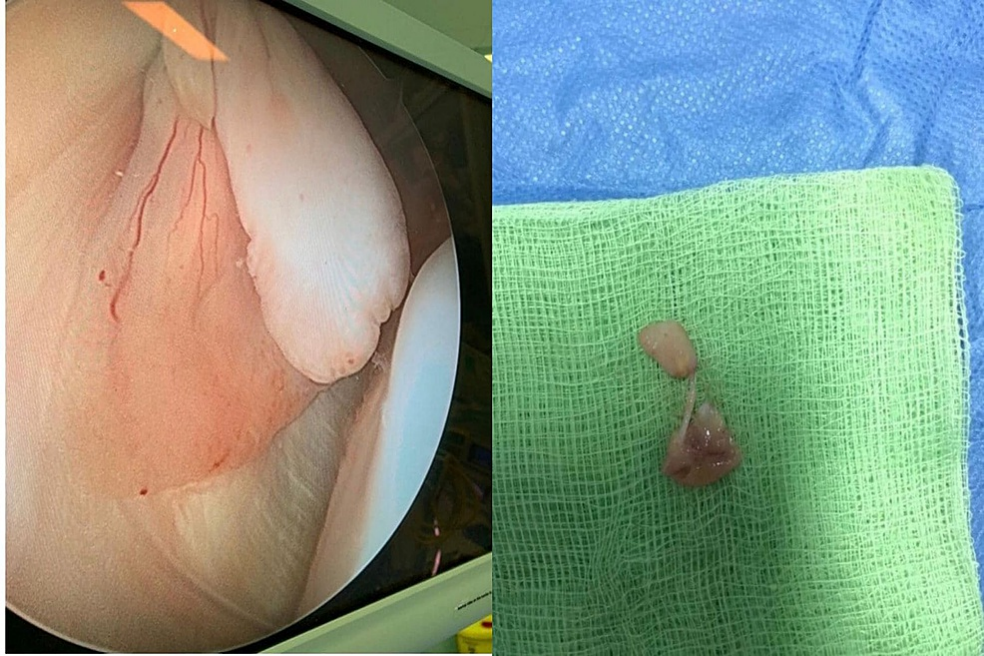
Intraoperative appearance of the lesion.

**Figure 4:**
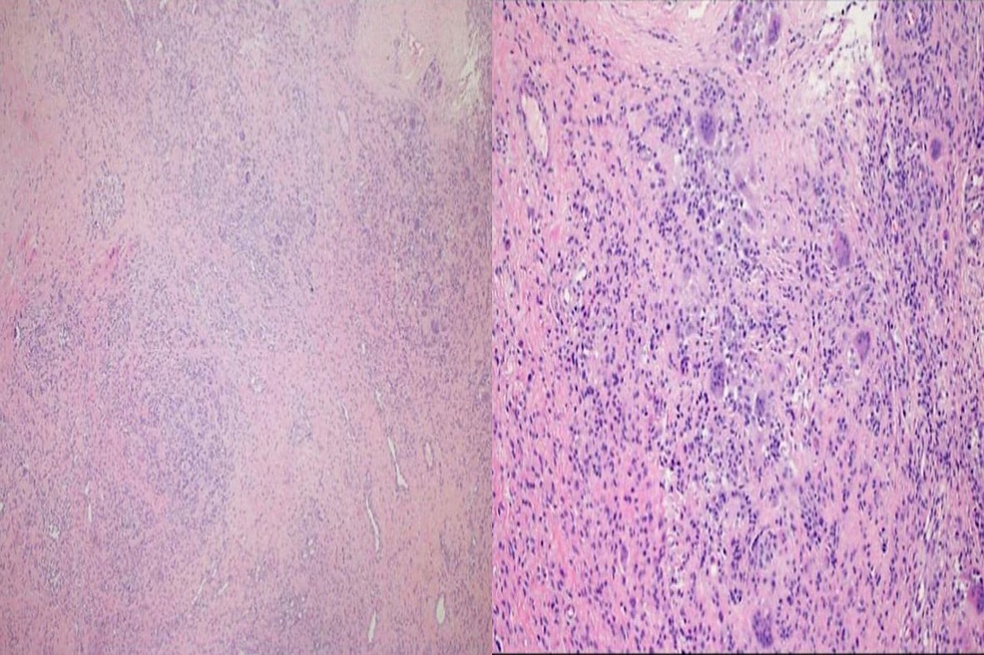
Histopathological appearance showed polymorphous population of cells, inflammatory cells along with multiple multinucleated giant cells.

The patient underwent arthroscopic excision of the lesion. There appeared to be a mass in the lateral gutter that was 3 cm x 2.4 cm x 0.4 cm in size, with a yellow-white, pedunculated, rubbery smooth surface. A whole excision with safe margins was completed (Figure 3). A histologic report showed a polymorphous population of cells with variable proportions of large histocytoid cells, osteoclast-like giant cells and smaller mononuclear stromal cells suggested of GCTTS (Figure 4). Scattered infiltrate along with few sclerotic collagenous areas was also noted, which lead to the diagnosis of GCTTS. Physical therapy was encouraged. No new local complaint or recurrence was detected over two years of postoperative follow-up.

## Discussion

A GCTTS is a benign lesion, arising from the synovial lining of membranes within joints, bursa, and tendon sheath. It is considered to be a localized form of a tenosynovial giant cell tumor, compared to PVNS which is diffuse and intra-articular [2, 6]. GCTTS is also found most commonly extra-articularly in the tendon sheath of the hand, which is the second most common pathology after a ganglion [4]. The knee joint is considered to be the most common site of involvement after the fingers, with mostly women between the ages of 30 and 50 affected [2, 4, 6]. The extra-articular form of a GCTTS presents with a painless mass gradually growing in the hands and feet. When compared to the intra-articular type, its presentation is often indolent and the patient may not have complaints until mechanical symptoms are noticeable [6-7].

Imaging is very important to aid in the process of diagnosis. Conventional radiography is the first step as a diagnostic tool in assessing and evaluating such vague and general knee symptoms. Most of the time, however, radiographs appear normal [6]. Thus, MRI is crucial in evaluating these cases. Although no typical appearance, the lesion usually appears ovoid in shape, is well marginated, and may or may not be pedunculated. T1-weighted images show slight hyperintensity in relation to skeletal muscles, and T2-weighted images show variable signal intensities due to hemosiderin deposits [7-8]. In our case, the differential diagnosis was assumed to be a ganglion. This conclusion was due to a combination of a benign history and a thorough examination and imaging. However, malignancy and other benign soft tissue tumors must be kept in mind.

A histologic examination is the definitive diagnostic workup necessary for establishing such a disease. The lesion is characterized by a fibrous stroma with a proliferation of the synovial tissue as villi or nodules, and the presence of irregular-shaped multinucleated giant cells within the hyaline stroma [9].

Surgical excision is considered the treatment of choice in a GCTTS, either with an arthroscopic approach or arthrotomy. The most common complication of treating such a case is the rate of recurrence, which is between 10% and 20%. The reason for this recurrence is that with inadequate excision, residual tumor cells are capable of regenerating. The rate of regeneration depends on how diffuse the lesion is in the knee, and how extensively it affects the joint [8, 10]. Functional outcomes are better with arthroscopic surgery compared to an open approach; however, doubt exists in tumor surgery principles [10-11].

## Conclusions

A GCTTS is a benign tumor arising from the synovial lining of tissues in joints, bursa, and tendon sheath. Although it is found dominantly in fingers, a GCTTS is not uncommon in other large joints such as the knee. Clinical history and presentation are of an indolent nature; thus MRI is the most optimal modality to outline the lesion and a histopathologic examination is the definitive diagnostic step. Total excision of the lesion is curative; however, cases of recurrence are possible with inadequate surgical methods.

The aim of the current study is to shed light on the fact that any intra-articular knee prominence should alert physicians of tumoral masses, which should put a GCTTS as a contender in the differential diagnosis.
